# Supplementary material for: Multi‐dataset identification of innovative feature genes and molecular mechanisms in keratoconus
Source: J Cell Mol Med. 2024 Sep 19;28(18):e70079. doi: 10.1111/jcmm.70079 (PMC11412914; doi:10.1111/jcmm.70079)
Supplement: Supplementary file 2 — Material S2. [file JCMM-28-e70079-s002.zip › 7.GSEA analysis/3.DBNDD1/KEGG_PRIMARY_IMMUNODEFICIENCY.html]

Details for gene set KEGG\_PRIMARY\_IMMUNODEFICIENCY[GSEA]

|  || Dataset | DBNDD1.group.cls#h\_versus\_l |
| Phenotype | group.cls#h\_versus\_l |
| Upregulated in class | h |
| GeneSet | KEGG\_PRIMARY\_IMMUNODEFICIENCY |
| Enrichment Score (ES) | 0.20211427 |
| Normalized Enrichment Score (NES) | 0.55505913 |
| Nominal p-value | 0.9940358 |
| FDR q-value | 0.99978405 |
| FWER p-Value | 1.0 |
Table: GSEA Results Summary

  

Fig 1: Enrichment plot: KEGG\_PRIMARY\_IMMUNODEFICIENCY      
 Profile of the Running ES Score & Positions of GeneSet Members on the Rank Ordered List

  

| SYMBOL | TITLE | RANK IN GENE LIST | RANK METRIC SCORE | RUNNING ES | CORE ENRICHMENT || 1 | IL7R | na | 2510 | 0.272 | 0.0040 | Yes |
| 2 | CD19 | na | 3104 | 0.247 | 0.0563 | Yes |
| 3 | CIITA | na | 3568 | 0.229 | 0.1071 | Yes |
| 4 | JAK3 | na | 5505 | 0.164 | 0.0974 | Yes |
| 5 | CD4 | na | 6459 | 0.138 | 0.1086 | Yes |
| 6 | CD8B | na | 6835 | 0.128 | 0.1337 | Yes |
| 7 | TNFRSF13C | na | 7128 | 0.122 | 0.1594 | Yes |
| 8 | CD3D | na | 8162 | 0.100 | 0.1578 | Yes |
| 9 | RAG1 | na | 8334 | 0.098 | 0.1803 | Yes |
| 10 | TNFRSF13B | na | 8984 | 0.086 | 0.1857 | Yes |
| 11 | AIRE | na | 9218 | 0.082 | 0.2021 | Yes |
| 12 | IL2RG | na | 10291 | 0.067 | 0.1901 | No |
| 13 | BLNK | na | 10880 | 0.060 | 0.1899 | No |
| 14 | CD3E | na | 11084 | 0.057 | 0.2001 | No |
| 15 | UNG | na | 12674 | 0.041 | 0.1658 | No |
| 16 | CD79A | na | 12880 | 0.039 | 0.1709 | No |
| 17 | RFXANK | na | 15546 | 0.019 | 0.0994 | No |
| 18 | PTPRC | na | 16868 | 0.010 | 0.0642 | No |
| 19 | DCLRE1C | na | 17991 | 0.003 | 0.0326 | No |
| 20 | AICDA | na | 19955 | -0.013 | -0.0204 | No |
| 21 | TAP1 | na | 20842 | -0.021 | -0.0402 | No |
| 22 | RFXAP | na | 21235 | -0.024 | -0.0447 | No |
| 23 | CD40 | na | 21562 | -0.028 | -0.0464 | No |
| 24 | TAP2 | na | 21858 | -0.031 | -0.0462 | No |
| 25 | RFX5 | na | 21866 | -0.031 | -0.0378 | No |
| 26 | IKBKG | na | 23063 | -0.044 | -0.0600 | No |
| 27 | ICOS | na | 24698 | -0.066 | -0.0884 | No |
| 28 | CD8A | na | 24920 | -0.070 | -0.0752 | No |
| 29 | IGLL1 | na | 25049 | -0.071 | -0.0589 | No |
| 30 | RAG2 | na | 27687 | -0.123 | -0.1004 | No |
| 31 | BTK | na | 28336 | -0.139 | -0.0800 | No |
| 32 | ZAP70 | na | 30432 | -0.202 | -0.0838 | No |
| 33 | LCK | na | 30718 | -0.211 | -0.0327 | No |
| 34 | CD40LG | na | 31492 | -0.240 | 0.0123 | No |
| 35 | ADA | na | 32418 | -0.288 | 0.0666 | No |
Table: GSEA details [plain text format]

  

Fig 2: KEGG\_PRIMARY\_IMMUNODEFICIENCY      
 Blue-Pink O' Gram in the Space of the Analyzed GeneSet

  

Fig 3: KEGG\_PRIMARY\_IMMUNODEFICIENCY: Random ES distribution      
 Gene set null distribution of ES for **KEGG\_PRIMARY\_IMMUNODEFICIENCY**
